# Nano-liposomal zein hydrolysate for improved apoptotic activity and therapeutic index in lung cancer treatment

**DOI:** 10.1080/10717544.2022.2057618

**Published:** 2022-04-01

**Authors:** Sahand Mazloum-Ravasan, Maryam Mohammadi, Elaheh Madadi Hiagh, Alireza Ebrahimi, Joo-Hyun Hong, Hamed Hamishehkar, Ki Hyun Kim

**Affiliations:** a Biotechnology Research Center, Tabriz University of Medical Sciences, Tabriz, Iran; b Department of Food Science and Engineering, Faculty of Agriculture, University of Kurdistan, Sanandaj, Iran; c Pharmaceutical Analysis Research Center, Tabriz University of Medical Sciences, Tabriz, Iran; d Research Center for Pharmaceutical Nanotechnology, Tabriz University of Medical Sciences, Tabriz, Iran; e Pediatrics III, University Hospital Essen, Essen, Germany; f Immunology Research Center, Tabriz University of Medical Sciences, Tabriz, Iran; g Drug Applied Research Center, Tabriz University of Medical Sciences, Tabriz, Iran; h School of Pharmacy, Sungkyunkwan University, Suwon, Republic of Korea

**Keywords:** Liposome, zein, hydrolysate, apoptosis, lung cancer

## Abstract

Lung cancer is one of the most common cancers in the world with a high mortality rate. Zein is a protein compound whose protein isolate is not useful and whose protein hydrolysis produces biological activity. By encapsulating this bioactive compound inside the nanoparticles (NPs), it causes itself to reach the tumor site and destroy it rapidly. In this study, the effects of zein hydrolysate (ZH) and nano-liposomal ZH (N-ZH) were investigated on the human A549 cell line. Western blotting and cell cycle analyses showed that ZH and N-ZH caused cytotoxicity. They induced apoptosis via cell cycle arrest at the G0 phase, as well as significant increases in pro-apoptotic genes, such as Bax, caspase-3, -8, -9, and p53, accompanied with significant decreases in the anti-apoptotic marker Bcl-2. Based on the results, the cytotoxic and anticancer effects of N-ZH were higher than those of free ZH. In conclusion, liposomes improved the performance of ZH and dramatically reduced the IC_50_ value of ZH. These findings provided the experimental evidence that N-ZH with favorable anticancer activity can be used as a therapeutic agent and strategy for lung cancer treatment in future clinical trials.

## 1. Introduction

Nowadays, the world suffers from cancer and the highest mortality rate in the world is related to lung cancer. The results show that 1.8 million people are diagnosed with lung cancer each year and 1.6 million people die from the disease (Ferlay et al., 2015; Carioli et al., 2021). Therefore, treatment of lung cancer patients in the world seems to be essential. Surgery, radiation therapy, chemotherapy and targeted therapy are currently used to treat lung cancer. However, the results of conventional therapies are not promising, except in local cancers (Otsuki et al., 2018). The main disadvantage of lung cancer chemotherapy drugs is that they are poorly distributed in the tumor tissue and do not specifically target tumor cells, circulating in the bloodstream in all parts of the body and leading to the death of healthy cells. Dose-limiting side effects are also limiting factors for this type of treatment (Mokwena et al., 2018).

Given that many strategies have been evaluated to treat tumor tissues and improve drug delivery to cancer cells, the use of nanotechnology is important for improving targeted drug delivery in cancer treatment (Sharma et al., 2019). Drug-loaded nanoparticles (NPs) increase the effect of their permeability on cancer cells and subsequently improve drug performance by increasing its amount in tumor tissue (Zhang et al., 2016). Also, one of the benefits of NPs treatment is that natural tissues are not exposed to them, so they will not have a negative effect on healthy cells. In addition, by increasing the accumulation of the drug in the desired location, the dose can be reduced and the incidence of side effects can be further reduced (McNeil, 2009). Lipid-based NPs are the main research focus owing to their safe and non-immunologic characteristics. Among the different types of lipid-based nanoparticles, liposomes are the most studied because of their advantages. Liposomes are entirely microscopic vesicles in which a volume of water is completely enclosed by a membrane composed of fat molecules (Rahimpour & Hamishehkar, 2012). As well as, liposomes allows the incorporation of a lipophilic drug in the lipid bilayers and a hydrophilic drug in the water chamber (Gharib et al., 2015). Recently, liposome-based drugs, e.g. amphotericin B, daunorubicin, cytarabine, morphine sulfate, vincristine, verteporfin, and nystatin, have been developed and even commercialized (Zylberberg & Matosevic, 2016; He et al., 2020).

Zein is obtained as a by-product of corn starch processing and so that 45-50% of corn protein is composed of it. Due to the negative nitrogen balance and poor solubility in water, zein isolate is not directly applicable for human consumption (Shukla & Cheryan, 2001). Also, zein is widely used in nano drug delivery systems due to its good biocompatibility (Yu et al., 2020). Protein hydrolysis improves the function of a protein by modifying the protein, which involves chemical and biological changes that alter the physicochemical and functional properties of the protein by changing the structure of the protein (Yalcin & Çelik, 2007). Zein hydrolysate (ZH) has been shown to possess many beneficial effects, including antihypertensive (Miyoshi et al., 1991), anti-inflammatory (Liang et al., 2018), and antioxidant (Tang et al., 2010) effects, and it can relieve or reduce the risk of many chronic diseases (Ashaolu, 2020).

Recent studies emphasize that liposomes are good carriers for the transfer of protein hydrolyzes (Jaspart et al., 2005) and by stabilizing them within their structure, they increase the half-life of protein hydrolyzes and subsequently increase their effectiveness in the circulatory system (Kaur et al., 2007). As far as we know, no study has been performed to compare the effect of ZH on lung cancer cells with nano-liposomal ZH (N-ZH), Therefore this study was pursued with the above goal. The results of this study may open new horizons in the application of ZH nanotechnology as a new natural-based compound in the treatment of cancer.

## 2. Materials and methods

### 2.1. Materials

Zein Protein (Merc, Germany), alcalase enzyme, lecithin, Tween 80, cholesterol, ethanol, RPMI, RNase A, and yellow tetrazolium 3-(4,5-dimethylthiazol-2-yl)-2,5 diphenyl tetrazolium bromide (MTT) was purchased from Sigma-Aldrich (St Louise, IL). Fetal bovine serum and DMSO were provided by Thermo (Waltham, MA), PBS, trypsin/EDTA, propidium iodide (PI), Triton X100, and DCFDA/H2DCFDA Cellular ROS Assay Kit were obtained from Abcam (Cambridge, UK). Penicillin, streptomycin, and Tris-HCl buffer were purchased from GeneDirex, Inc. (Gongye Rd, Taiwan). Annexin V-FITC (EXBIO, Czech), Bax, Bcl-2, caspase-3, -8, -9, and p53 antibodies were purchased from Santa Cruz Biotechnology (Santa Cruz, CA, USA).

### 2.2. Preparation of ZH

Zein suspension (5% *w/v*) was hydrolyzed with alcalase at pH 9 in a 50 °C shaker incubator for 4 h. The enzyme-to-substrate mass ratio was 2.5% *w/v.* During hydrolysis, the pH of zein suspension was maintained at 9.0 by adding 1 M NaOH. After 4 h of hydrolysis, the pH of the hydrolysate was reduced to 7.0 by adding 1 M HCl, and the enzyme was inactivated by heating the mixture at 85 °C for 10 min. Next, the suspension was centrifuged at 5000 × *g* for 10 min. The hydrolysate was collected and freeze-dried for further analysis (Wang et al., 2014).

### 2.3. Liposome preparation

Liposomes were prepared via the thin layer hydration-ultrasound method. To a round balloon containing 10 mL of absolute ethanol as a solvent, soy lecithin and cholesterol were added at a ratio of 90:10 (*w/w*). Using a rotary evaporator at 40 °C, the solvent was removed from the mixture, and a thin layer was prepared. The resulting film was hydrated by 5 mL of water containing 10 mg of ZH and 10 mL of 1X PBS solution. To reduce the size of the liposomes, the formulation was placed in an ice bath for 10 min (10 cycles, 1 min ultrasound) with an ultrasound probe (130 watts, 20 kHz; Vibra Cell Sonics & Material, USA) at 80% power (Mohammadi et al., 2014).

### 2.4. Characterization of N-ZH

The drug loading (DL) and encapsulation efficiency (EE) percentages were determined by measuring the concentration of unloaded ZH in liposomes. Briefly, 2 mL of each sample was placed into the Amicon (Ultra-4 100 k–a 30 kDa) filter and centrifuged at 4000 rpm for 10 min. Next, by UV-vis spectrophotometry (Ultrospec 2000 UV/Visible; Pharmacia Biotech Instruments, Ltd., Cambridge, England), the concentration of unloaded ZH was determined at 278 nm. EE was calculated using Equation (1) to determine the concentration of encapsulated ZH, and DL was calculated according to Equation (2) using the concentration of ZH loaded into nanoliposomes estimated from EE, which was inserted in the numerator, and the total weight of nanoliposomes, which was inserted in the denominator of Equation (2) (Nazari et al., 2019):

(1) $$\mathrm{EE}(\%)=\frac{{\mathrm{ZH}}_{\mathrm{total}}-{\mathrm{ZH}}_{\mathrm{unloaded}}}{{\mathrm{ZH}}_{\mathrm{total}}}\times 100$$

(2) $$\mathrm{DL}(\%)=\frac{{\mathrm{ZH}}_{\mathrm{loaded}}}{\mathrm{Nanoliposome}}\times 100$$

### 2.5. Size and zeta potential (ZP) analyses

Nanoliposome size and ZP were measured at room temperature using dynamic light scattering and a Malvern zeta sizer (Malvern, UK). All samples were diluted 10 times with distilled water, and all measurements were performed in triplicate.

### 2.6. MTT assay

To evaluate the cytotoxic effect of ZH and N-ZH on A549 cells, the cells (10^4^ cells in each well) were incubated in 96-well plates for 24 h. After 24 h, the cells were treated with ZH and N-ZH at concentrations of 1, 2, 4, 8, 16, 32, 64, 128, 256, 512, and 1024 μg/mL for 48 h. The cells were then incubated with MTT (5 mg/mL in PBS) for 4 h at 37 °C. The supernatant was discarded, and the cells were incubated with 200 μL of DMSO for 20 min, and finally measured at 570 nm using an ELISA Reader (Mazloum-Ravasan et al., 2020).

### 2.7. Apoptosis analysis

#### 2.7.1. Annexin V/PI assay

Apoptosis rate of A549 cells was evaluated using an Annexin V-FITC kit (EXBIO, Czech) and flow cytometry. In summary, A549 cells (10^6^ cells in each well) were incubated for 24 h. The cells were incubated with ZH and N-ZH suspension at the IC_50_ dose for 48 h. The cells were then removed from the wells and washed twice with PBS. For every 100 μL of cell sample, 10 μL of 50 mg/μL PI solution and 5 μL of 25 mg/μL FITC-conjugated Annexin V solution were added, and the cells were kept in darkness for 20 min. Samples were analyzed by flow cytometry on a MACS Quant instrument. (Vermes et al., 1995).

#### 2.7.2. Cell-cycle distribution

Cells (10^6^) treated with ZH and N-ZH were washed with PBS and fixed for 24 h at −20 °C in 70% cold ethanol. After 24 h, the cells were washed with PBS and incubated with 10–50 μg/mL RNase A and 20 μg/mL PI at 37 °C in darkness for 20 min. Finally, they were analyzed by flow cytometry (Vanzyl et al., 2018).

#### 2.7.3. Intracellular reactive oxygen species (ROS) assay

Cells (10^6^) were treated with the IC_50_ dose of ZH and N-ZH for 48 h. Afterward, the cells were washed with PBS and incubated with 2-7-dichlorodihydrofluorescein diacetate (DCFH-DA) for 40 min at 37 °C. The cells were subsequently washed twice with PBS. ROS levels in the cells were measured by flow cytometry (Mazloum-Ravasan et al., 2020).

#### 2.7.4. DAPI staining

DAPI staining was conducted to identify apoptotic cells showing signs of nuclear chromatin compaction and fragmentation. According to the manufacturer’s instructions, the cells were cultured for 48 h with ZH and N-ZH and then stained with DAPI staining solution. Cell images were obtained by fluorescence microscopy (Leica, Wetzlar, Germany). The percentage of apoptotic cells was calculated by the Image-Pro Plus 6.0 software (Wang et al., 2020).

#### 2.7.5. Wound healing assay (scratch assay)

The effect of N-ZH and ZH on the migration capabilities of A549 cells was evaluated using the scratch wound method. Cells (3 × 10^5^) were cultured in a 12-well plate. Next, using a plastic pipette tip, a thin scratch was created in the cell layer, followed by treatment with N-ZH and ZH at the IC_50_ dose. At 0, 12, and 24 h, photographs of the cells were taken (Mazloum-Ravasan et al., 2021).

#### 2.7.6. Western blotting assay

In 6-well plates, 4 × 10^5^ A549 cells were incubated for 24 h. The cells were then treated with ZH, and N-ZH for 48 h. After washing twice with PBS, the cells were placed in a lysis buffer for 30 min and suspended. The suspended cells were centrifuged at 14,000 rpm for 20 min. The supernatant was collected, and the protein content was measured through the Bradford method. Protein lysates were separated by 12% SDS gel electrophoresis and then transferred to a PVDF membrane. The membrane was then placed in a blocking solution (2% skim milk in TBS buffer) for 1 day. After that, Bax, Bcl-2, caspase-3, 8, 9, and p53 antibodies were mixed with the blocking solution, and the membrane was further incubated for 16 to 18 h. After initial staining of the membrane with secondary rabbit antibody with concentration (1: 1000) for all primary antibodies for one hour and 15 min at room temperature. The GAPDH antibody was used as a loading control (Shirjang et al., 2020).

### 2.8. Statistical analysis

All data results were analyzed using GraphPad Prism (version 8.0). *p* < .05 was considered statistically significant. The data were analyzed to determine the difference in means between groups.

## 3. Results

### 3.1. Preparation and characterization of N-ZH

The liposome size was calculated to be 43.44 ± 11.8, with an −2.25 ± 0.37 ZP and 0.184 PDI. Moreover, the EE and DL of the liposome formulation were calculated as 94.6 ± 3.1 and 9.46 ± 0.31, respectively.

### 3.2. Cell proliferation assay

**Figure 1.**
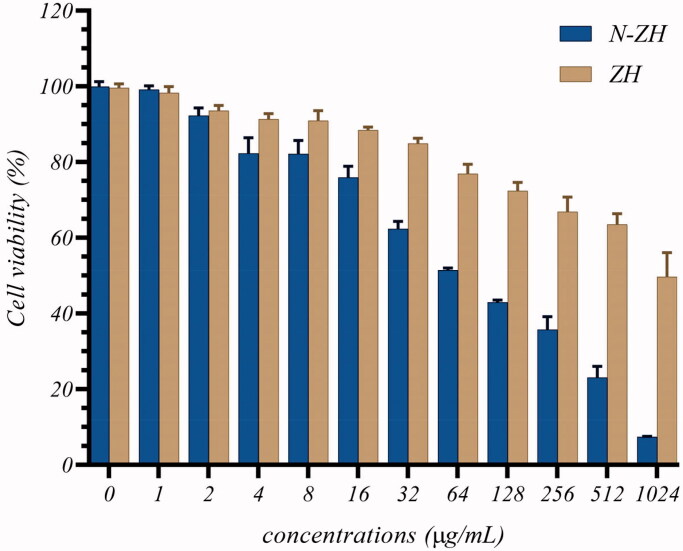
Effect of ZH and N-ZH treatment for 48 h on A549 cell growth was determined by MTT assay. The results were expressed as the percentage of living cells compared to those in the control group (0 μg ZH and N-ZH). Zein hydrolysate (ZH), Nano-liposomal ZH (N-ZH).

The IC_50_ value of N-ZH and ZH was calculated to be 61 and 864 μg/mL, respectively, against A549 cells after 48 h of treatment. These results suggested that ZH and N-ZH were cytotoxic to A549 human cancer cells, with N-ZH showing 14 times higher cytotoxicity than ZH (Figure 1).

### 3.3. Results of apoptosis analysis

#### 3.3.1. Annexin V/PI assay

**Figure 2.**
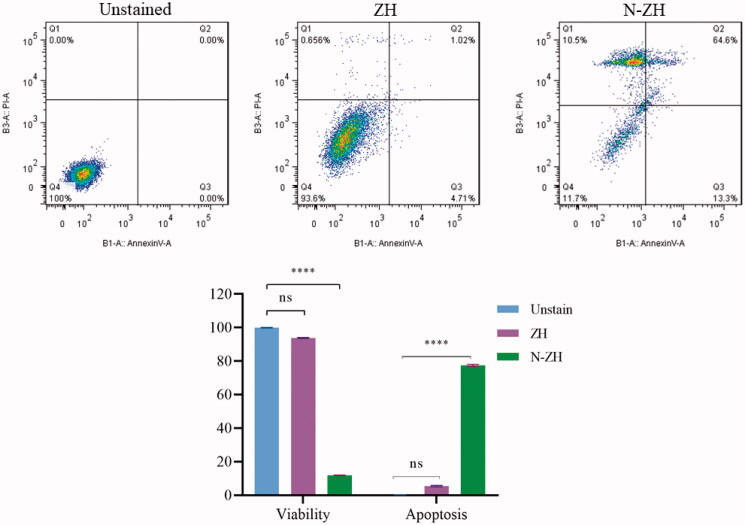
Effects of ZH and N-ZH treatment at the IC_50_ dose on A549 cell apoptosis were examined using Annexin-V/PI staining and flow cytometry (****p < .0001). Zein hydrolysate (ZH), Nano-liposomal ZH (N-ZH), Nonsignificant (ns).

Apoptosis in N-ZH- and ZH-treated cells was examined by assessing the binding of fluorescent Annexin V. Exposure to N-ZH and ZH promoted the staining of A549 cells with fluorescent Annexin V (Figure 2). Specifically, treatment with N-ZH and ZH at 61 μg/mL significantly (****p < .0001) increased Annexin V upregulation. N-ZH inhibited cell proliferation, and but promoted cell apoptosis, as indicated by the increased fluorescence intensity of Annexin V (Figure 2).

#### 3.3.2. Cell-cycle distribution

**Figure 3.**
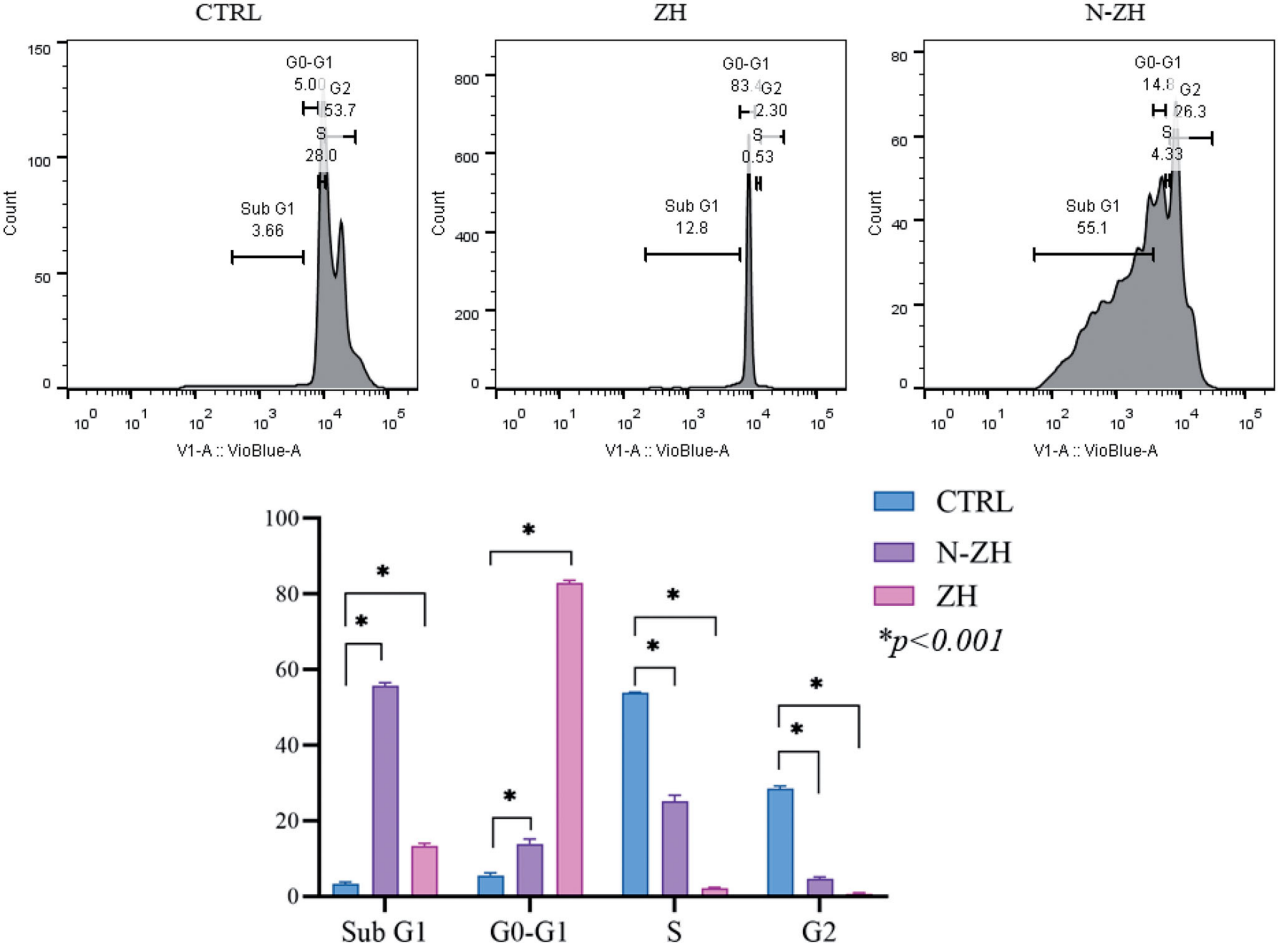
Effect of ZH and N-ZH treatment at the IC_50_ dose on the cell cycle of A549 cells was determined after 48 h. (*p < .001). Zein hydrolysate (ZH), Nano-liposomal ZH (N-ZH).

N-ZH led to cell-cycle arrest in A549 cells. As shown in Figure 3, treatment with N-ZH caused a significant decrease in the percentage of cells in the S phase and G2/M phase compared to the control cells (*p < .001). The rate of cells in the Sub-G1 phase was significantly increased among N-ZH-treated cells compared to that among control cells (*p < .001).

#### 3.3.3. Intracellular reactive oxygen species assay

**Figure 4.**
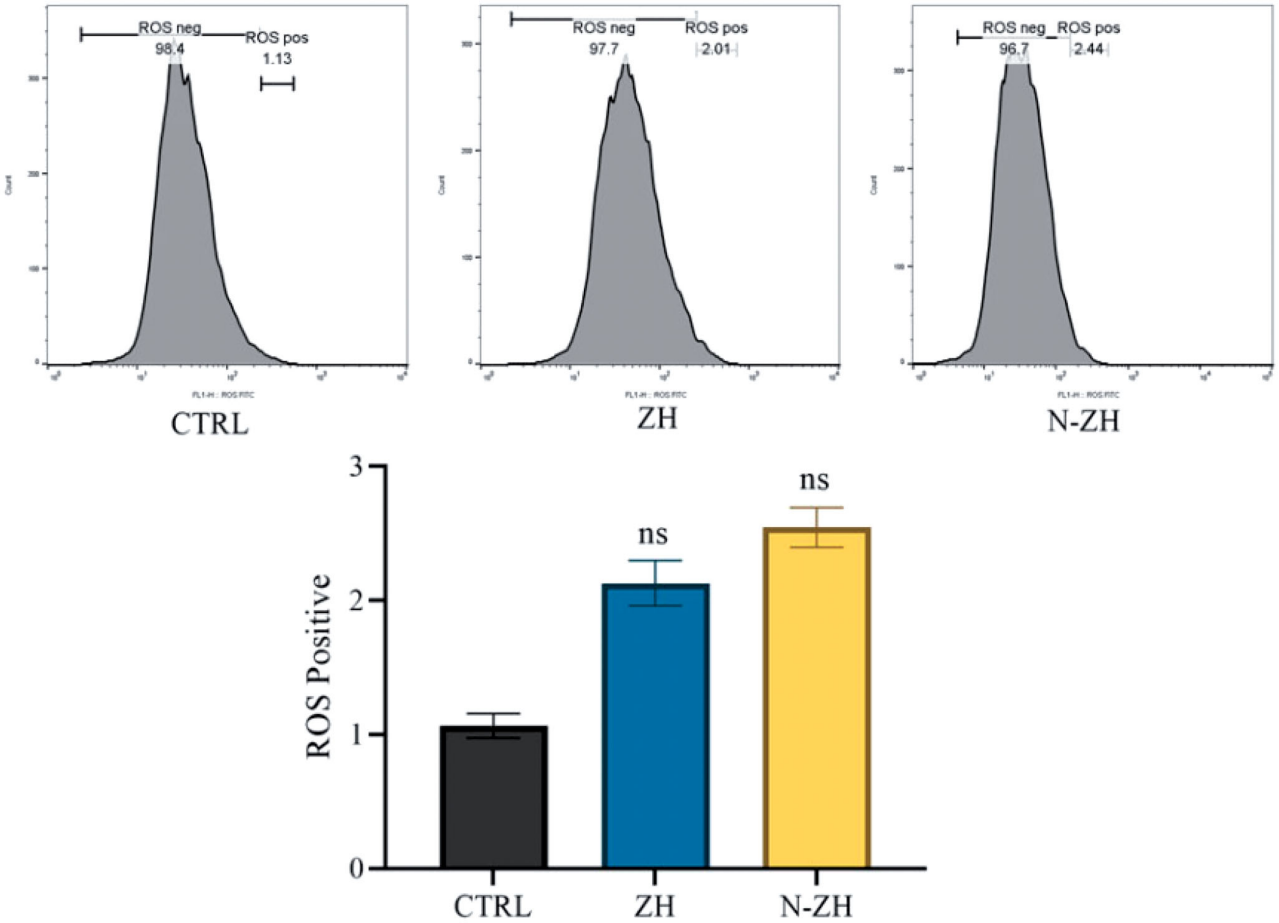
Effect of ZH and N-ZH treatment at the IC_50_ dose on ROS levels was measured at the cellular level after 48 h. Zein hydrolysate (ZH), Nano-liposomal ZH (N-ZH) Nonsignificant (ns).

ROS have previously been shown to mediate intracellular signaling cascades and stimulate programmed cell death pathways (Kobori et al., 2007). The current results showed that treatment with 61 μg/mL N-ZH did not cause a significant change in the intensity of DCF fluorescence in A549 cells (Figure 4).

#### 3.3.4. DAPI Staining

**Figure 5.**
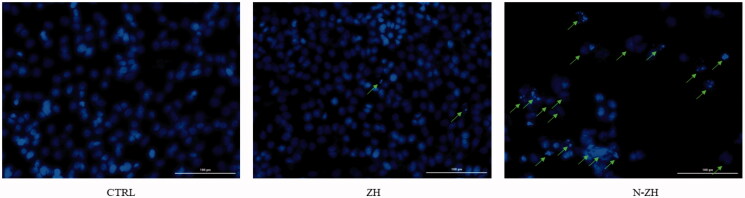
Morphological alterations in cell nuclei during ZH- and N-ZH-induced apoptosis in A549 cells, observed by DAPI staining and fluorescence microscopy. Scale bar, 100 µm. Zein hydrolysate (ZH), Nano-liposomal ZH (N-ZH).

DAPI staining was performed to evaluate the effect of ZH and N-ZH on nuclear changes in A549 cancer cells. Significant morphological changes were observed in the cell structure and the cell nucleus of the ZH and N-ZH groups compared to the control group. The ZH- and N-ZH-induced changes in the nuclear morphology led to apoptosis in cancer cells (Figure 5). The number of apoptotic cells in the N-ZH-treated group was higher than that in the ZH group, consistent with the MTT assay results.

#### 3.3.5. Wound healing assay (scratch assay)

**Figure 6.**
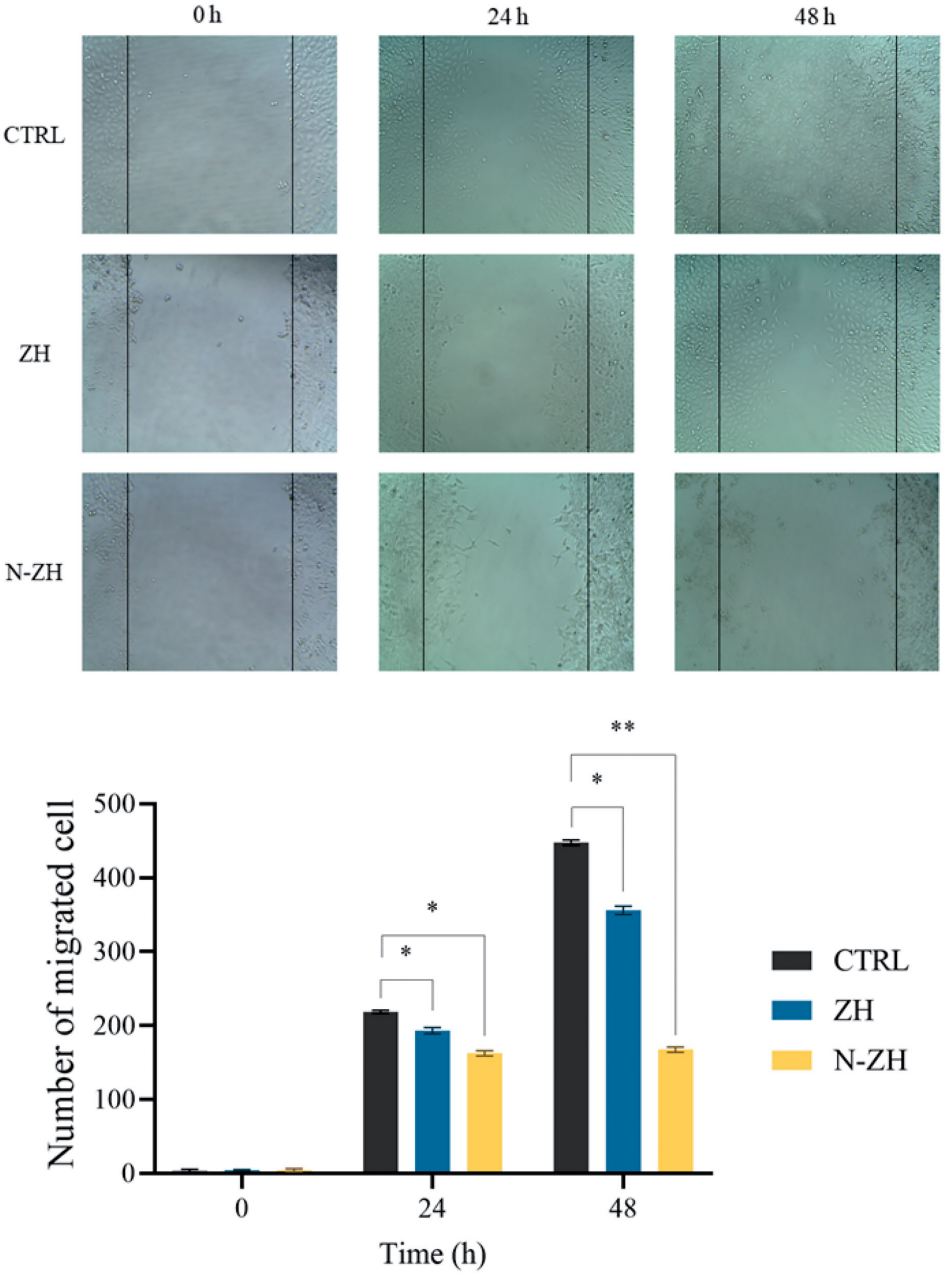
N-ZH attenuates the migration of A549 cells. The healing ability of A549 cells in N-ZH-treated group was significantly weaker than that in the control and ZH-treated groups at 24 and 48 h after the wound was scratched. *p < .05 and **p < .01. Zein hydrolysate (ZH), Nano-liposomal ZH (N-ZH).

The migration of A549 cells was investigated using the scratch method. As shown in Figure 6, N-ZH-treated cells migrated a significant distance compared to the control, and N-ZH inhibited the migration of A549 cells. The difference was significant compared to the control and ZH groups (**p < .01).

#### 3.3.6. Western blotting assay

**Figure 7.**
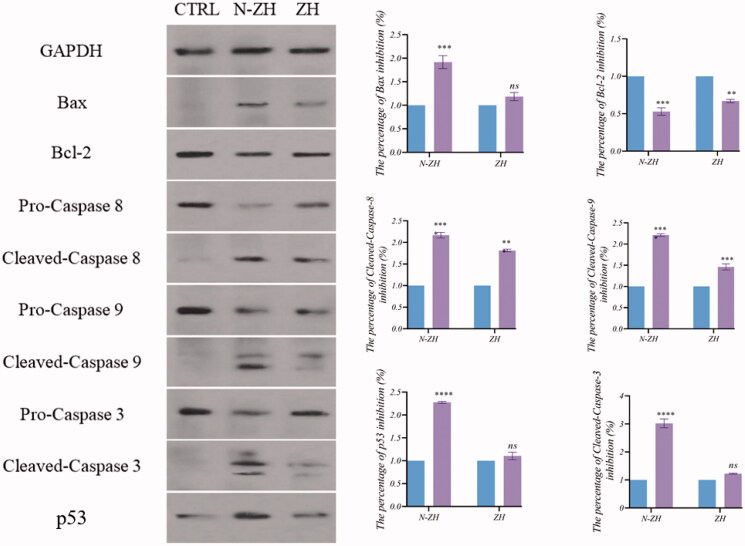
Effects of ZH and N-ZH treatment at the IC_50_ dose on the expression of apoptosis-related proteins, as determined by western blotting. Markers of apoptosis in A549 cells include caspase 8, caspase 3, caspase 9, p53, and Bcl-2-to-Bax ratio. GAPDH was used as the internal control. ***p < .001 and ****p < .0001 vs. the 0 µg group. Zein hydrolysate (ZH), Nano-liposomal ZH (N-ZH) Nonsignificant (ns).

The expression levels of apoptosis-related proteins were measured in A549 cells. After N-ZH treatment, the expression levels of caspase-8 (****p < .0001), caspase-9 (****p < .0001), caspase-3 (****p < .0001), and p53 (****p < .0001) increased, whereas the ratio of Bcl-2 to Bax (***p < .001) decreased. These data suggested that N-ZH induced apoptosis in A549 cells via the mitochondrial and external pathways (Figure 7).

## 4. Discussion

Apoptosis is a process of cell death that occurs in a planned manner. Several drugs induce DNA damage through two critical pathways that regulate mitochondrial mediators (internal pathway) and the external pathway (Elmore, 2007). Therefore, promoting the repair and apoptosis of cancer cells is a promising approach to cancer treatment (Fesik, 2005). Previous studies have shown that bioactive substances derived from natural products exert antitumor effects by inducing innate apoptosis (Chou et al., 2010; Sun et al., 2013).

The antitumor activity of ZH was determined via an MTT assay. Compared to egg white protein hydrolysate (Moon et al., 2017), N-ZH killed 50% of cancer cells at a much lower concentration. Moreover, the MTT assay revealed that N-ZH exhibited significantly improved anticancer activity compared with ZH. Flow cytometry analysis results showed that ZH increased the percentage of early and late apoptotic cells in A549 cells, and similar results have been obtained with silkworm pupa protein hydrolysate (Li et al., 2018). The above findings indicated a significant (****p < .0001) improvement in the apoptosis-inducing effect of N-ZH at much lower concentrations compared to ZH. Hsieh et al. analyzed the antitumor activity of soy protein extract on rat fibroblasts and reported antitumor activity. Other studies have reported the anticancer activity of turnip flour-derived peptides (Hsieh et al., 2010). Inhibitory activity in cervical cancer cell growth (HeLa) was reported by Li (Li et al., 2013). Also found induction of cell death in cells exposed to a fraction of seed-derived peptides. Explains the possible mechanisms by which plant-derived peptides have antitumor or anticancer activity. This indicates that the hydrolysis process, increases the anti-proliferative capacity of certain proteins derived from grains, including saddle. Immunofluorescence analysis by DAPI staining showed changes in the morphology of the nucleus after treatment: ZH-treated cells showed marked apoptotic changes such as round cell lines, low cytoplasm containing some granules, and cytoplasmic membrane blisters in the cell, while N-ZH showed more strongly wrinkled, dense, and fragmented nuclei, indicating varying degrees of apoptosis (Tang et al., 2010). Isolated nuclei with normal heterochromatin density, nuclear disruption was observed by nucleus-vesicles (Figure 2), which is the result of the expression of apoptotic factors including caspases and Bcl-2 family due to zein hydrolysis activity. Based on a previous study (Berköz et al., 2020) induction of apoptosis by hydrolysis of Roe protein showed morphological changes in cancer cells. Following the study of cell apoptosis due to ZH and according to the results of flow cytometry and Annexin V test, ZH causes apoptosis in A549 cancer cells. The rate of apoptosis in cells is dramatically increased by encapsulating ZH. The Bcl-2 family plays an important role in initiating the innate apoptotic pathway (Gogvadze et al., 2006). In this study, Western blot analysis confirmed that ZH suppresses the expression of anti-apoptotic protein Bcl-2 and promotes Bax expression of pro-apoptotic protein. The results also showed that apoptosis is activated through both internal and external pathways. Caspase activation was closely related to mitochondrial outer membrane permeability regulated by members of the Bcl-2 family, Zein hydrolysis induces the expression of genes associated with internal (caspase-9) and external (caspase-8) and caspase-3 apoptosis as functional caspases in A549 cells, which causes apoptosis and cancer cell death (Figure 7). Increased expression of Bcl-2 leads to resistance to chemotherapy and radiation therapy, while decreased expression of Bcl-2 may increase the apoptotic response to anticancer drugs. Also, the results showed that ZH treatment altered the transcriptional expression of genes directly involved in apoptosis, and that apoptosis-related pathways fluctuated after ZH treatment. In addition, p53 is a tumor suppressor, a transcription factor regulating downstream genes in cell cycle arrest, DNA repair, and apoptosis, affecting a range of cellular functions, including cell cycle regulation (Meek, 2015). Loss of p53 in many cancer cells leads to genomic instability, cell cycle dysfunction, and inhibition of apoptosis. After DNA damage, p53 kept the cell at a checkpoint to repair the damage. If the damage was irreversible, apoptosis would occur. In this study, expression of p53 by ZH was increased in a dose-dependent manner, confirming that ZH suppresses tumorigenesis and induces apoptosis. Comparison of ZH and N-ZH showed that nano-liposomes improve ZH function and allow ZH to activate apoptotic pathways at lower doses. Compared to a study on silkworm pupal hydrolysate protein (Li et al., 2018), the expression of apoptosis-related genes was higher in the present study. In cancer cells, ROS level increases as cellular metabolic activities, including messaging pathways or mitochondrial dysfunction, increase (Bhatia et al., 2016). However, in this study, ROS levels did not differ significantly between A549 cells treated with different doses of ZH and N-ZH. In contrast, Xue et al. (2015) showed that pea peptides hydrolysate reduces ROS levels in MCF-7 and MDA-MB-231 cells. According to Torres-Fuentes et al. (2015) and Zhang et al. (2018), chickpea and soy protein hydrolysates exert antioxidant properties in Caco2 cells. In cell cycle arrest, tumor suppressors or protooncogene activation can increase the dependence of cancer cells on G1-phase cyclin-dependent kinases in the S/G2/M stages, as well as DNA damage and exacerbation (Sherr & Bartek, 2017). In this study, we found that ZH induced cell cycle arrest in the G1 phase (Figure 3) (Tang et al., 2007). ZH also reduced the proportion of cells in the S and G2/M phases compared to the control, but elevated the proportion of cells in the G0-G1 and sub-G1 stages (when the cells underwent apoptosis) (Figure 3). Therefore, the cell cycle arrest induced by ZH treatment could be related to p53. It is thought that these p53 proteins activated by ZH may inhibit the complex between cyclin D1 and CDK4 protein required for retinoblastoma protein phosphorylation in G1/S. Moreover, N-ZH showed a higher efficacy than free ZH in inducing cell cycle arrest in the sub-G1 phase. The anti-lung cancer efficacy of sea hare hydrolysate (Nyiramana et al., 2020) was higher than that reported by Xiaotong Li et al. (2018). Examination of cell migration using the scratch method also showed that ZH and N-ZH treatment significantly inhibited cell migration at 24 and 48 h. This effect of ZH in inhibiting cell migration was significantly improved through encapsulation in nanoliposomes (**p < .01). Cancer cell migration was also inhibited in a previous study (Nyiramana et al., 2020), but the efficacy of corn hydrolysate was higher.

Liposomes play a significant role in enhancing drug delivery in many areas of biomedicine. Liposomes can stabilize therapeutic compounds, overcome cellular and tissue absorption barriers, and elevate the distribution of compounds at the target sites *in vivo*. Liposomes allow efficient delivery of encapsulated compounds to the target sites while minimizing systemic toxicity. In the present study, the liposomes were in the appropriate diameter size range. The ideal characteristics of liposome systems include a size below 100 nm, low PDI, and good dispersion. Liposomal EE and DL were higher for ZH, indicating good encapsulation efficiency and good ZH loading within liposomes. Compared to nanoparticles in a previous study (Hong et al., 2018), the ZH-loaded nanoparticles in the present study were smaller in size, had lower zeta potential, and had higher loading capacity. Nanocarriers can deliver drugs to the tumor site, thereby reducing their side effects and evading inactivation by the immune system, further increasing the therapeutic effectiveness. Liposomes are not effective in transporting liposomal drugs, despite their known potential for all tumors (Blenke et al., 2013; Amjadi et al., 2020). Liposomes can escape from the reticuloendothelial system and remain in the circulatory system for a long time, which maintained their specific tumor targeting effectiveness *in vivo* (Torchilin, 2005). Nevertheless, it should be noted that many research have shown disappointing results, some of which are related to the nano-medical formulations themselves. Therefore, in addition to designing various nanocarriers, we must also focus on developing strategies to overcome this high diversity in EPR and EPR monitoring and modification methods to increase the chances of clinical success (Theek et al., 2016).

**Figure 8.**
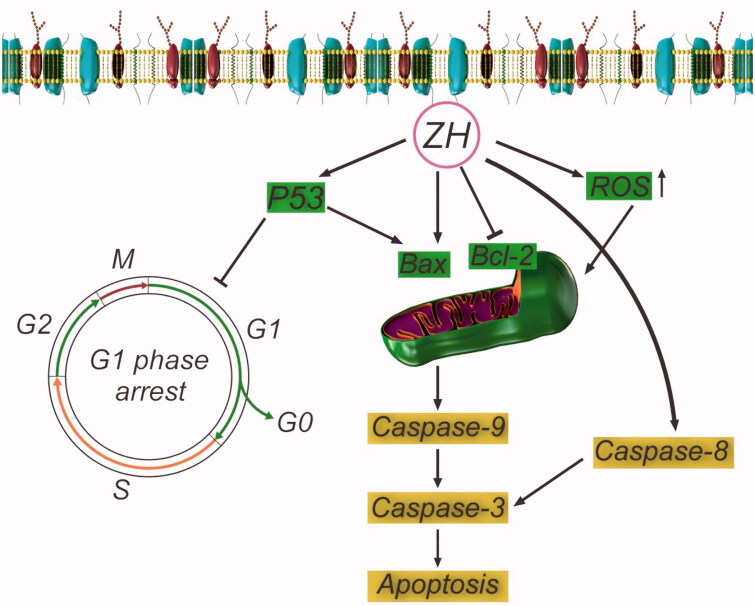
Pathways of apoptosis induction and cell cycle arrest by ZH.

Taken together, the findings of this study showed that ZH significantly increased A549 cell proliferation by increasing the expression of genes effective in inducing internal and external apoptosis including caspases-3, -8, -9 and P53 and the important Bcl-2 family and stopping It suppresses the cell cycle in phase S (Figure 8). It also significantly inhibited the migration of A549 cells. Moreover, the use of nanoliposomes significantly improved the antitumor effects of ZH, allowing ZH to induce apoptosis at much lower doses. Today, antitumor drugs with excellent safety and efficacy are essential because several anticancer drugs in clinical applications can cause harmful side effects on normal cells (Gutzmer et al., 2012). Our results suggest the potential use of ZH in the specific therapeutic management of lung cancer and the benefits of liposomal nanoparticles for improving the performance of anticancer drugs.

## 5. Conclusion

In this study, we found that ZH exposure activated cell apoptosis pathways. ZH-loaded liposomes were successfully synthesized in this study. These liposomes improved the anticancer effect of ZH, inducing apoptosis even at low doses. These findings show that N-ZH can inhibit the survival and migration of cancer cells.
